# CsMYB Transcription Factors Participate in Jasmonic Acid Signal Transduction in Response to Cold Stress in Tea Plant (*Camellia sinensis*)

**DOI:** 10.3390/plants11212869

**Published:** 2022-10-27

**Authors:** Zhaolan Han, Chen Zhang, Huan Zhang, Yu Duan, Zhongwei Zou, Lin Zhou, Xujun Zhu, Wanping Fang, Yuanchun Ma

**Affiliations:** 1 College of Horticulture, Nanjing Agricultural University, Nanjing 210095, China; 2 Department of Biology, Faculty of Science, Wilfrid Laurier University, Waterloo, ON N2L 3C5, Canada; 3 Forestry and Pomology Research Institute, Shanghai Academy of Agricultural Sciences, Shanghai 201403, China

**Keywords:** *Camellia sinensis*, MYB TFs, MeJA, cold stress response, interaction

## Abstract

Low-temperature stress is an increasing problem for the cultivation of tea (*Camellia sinensis*), with adverse effects on plant growth and development and subsequent negative impacts on the tea industry. Methyl jasmonate (MeJA), as a plant inducer, can improve the cold-stress tolerance in tea plants. R2R3-MYB transcription factors (TFs) are considered potentially important regulators in the resistance to cold stress in plants. However, the molecular mechanisms, by which MYB TFs via the jasmonic acid pathway respond to cold stress in the tea plant, remain unknown. In this study, physiological and biochemical assays showed that exogenous MeJA application could effectively promote ROS scavenging in the tea plant under cold stress, maintaining the stability of the cell membrane. Sixteen R2R3-MYB TFs genes were identified from the tea plant genome database. Quantitative RT-PCR analysis showed that three *CsMYB* genes were strongly induced under a combination of MeJA and cold-stress treatment. Subcellular localization assays suggest CsMYB45, CsMYB46, and CsMYB105 localized in the nucleus. Exogenous MeJA treatment enhanced the overexpression of *CsMYB45*, *CsMYB46*, and *CsMYB105* in *E. coli* and improved the growth and survival rates of recombinant cells compared to an empty vector under cold stress. Yeast two-hybrid and bimolecular fluorescence complementation experiments confirmed that CsMYB46 and CsMYB105 interacted with CsJAZ3, CsJAZ10, and CsJAZ11 in the nucleus. Taken together, these results highlight that *CsMYB45*, *CsMYB46*, and *CsMYB105* are not only key components in the cold-stress signal response pathway but also may serve as points of confluence for cold stress and JA signaling pathways. Furthermore, our findings provide new insight into how MYB TFs influence cold tolerance via the jasmonic acid pathway in tea and provide candidate genes for future functional studies and breeding.

## 1. Introduction

The tea plant (*Camellia sinensis* (L.) O. Kuntze) is an economically important perennial evergreen woody crop distributed in tropical to temperate regions [1]. In recent years, because of the frequent occurrence of extreme weather, cold stress has become one of the most destructive abiotic stresses in tea-plant cultivation. For example, a sudden frost in early spring will cause injury in and even death of tea buds and leaves. Therefore, cold stress is one of the crucial factors that affects tea plants’ growth and development and has subsequent negative impacts on the tea industry [1]. In natural stresses such as low temperature events, preventive measures by humans such as optimized cultivation measures and improving cultivation conditions have mainly been adopted in tea-plant production. In particular, introducing exogenous plant hormones can improve stress resistance and is one of the most effective strategies to enhance cold resistance in tea plants. Plant hormones such as jasmonic acid (JA), salicylic acid (SA), and ethylene promote the transmission of endogenous signal transduction substances in plants, regulate plant metabolism, and activate the plants’ immune and growth systems against abiotic stress. JA is one of the more widely used plant hormones and has been shown to play a positive role in alleviating the cold stress in many plants.

Jasmonates (JAs) are a type of lipid-derived phytohormone, which include jasmonic acid and its derivatives, such as methyl jasmonate (MeJA), jasmonoyl-isoleucine (JA-Ile), and 12-OH-JA [2]. MeJA regulates multiple plant processes, including organ development, cell cycle regulation, secondary metabolite biosynthesis, and defense responses to various biotic and abiotic stresses [3,4]. Increasing evidence has shown that MeJA plays a positive role in inducing cold stress tolerance in horticultural crops, such as mango [5], banana [6], kiwi [7], and pitaya [8]. Cold exposure rapidly induces the expression of JA biosynthesis-related genes, among which jasmonate-ZIM-domain (JAZ) proteins are important repressors of the JA signaling pathway. JAZ mediates various JA-regulated secondary metabolic processes, including the flavonoids’ biosynthesis through the interaction with various transcription factors (MYB/MYC/WRKY/bHLH/COI). In particular, JA-signaling-mediated anthocyanin biosynthesis is mainly regulated by the transcriptional level through R2R3-MYB TFs, in response to abiotic stress in different plants [9]. Studies have shown that *MYB* genes are induced by environmental factors such as temperature, exogenous hormones, and light and regulate anthocyanin biosynthesis [10,11,12,13].

The MYB transcription factor family is widely presented in all eukaryotes, and they are functionally diverse and constitute one of the largest protein families in plants. MYB TFs contain one to four highly conserved MYB DNA-binding domain repeats (R), which are classified into the 1R, R2R3, R1R2R3, and 4R MYB subfamilies. The R2R3 MYB TFs are the largest MYB subfamily and are involved in various processes, including primary and secondary metabolism, development, hormone regulation pathways, and responses to biotic and abiotic stresses [14]. Different studies have suggested that members of the MYB family participate in plant responses to temperature stress, which have been confirmed in numerous species such as *Arabidopsis* [14], maize (*Zea mays*) [15], rice (*Oryza sativa*) [16], soybean (*Glycine max Merri*) [17], apple (*Malus domestica*) [18], and tea plant (*Camellia sinensis*) [19]. Chen et al. identified and analyzed 122 members of R2R3-MYB family in tea plants and showed that MYB members have different responses to cold and drought stress, plant hormones (Gibberellins and Abscisic acid) [19]. Overexpression of *MdMYB23* improved the cold tolerance in transgenic apple calli and *Arabidopsis* [20], while overexpression of *GmMYB76* and *GmMYB177* enhanced the cold tolerance of soybeans. A previous study indicated that overexpression of *SIGMYBL2* improved cold resistance in tobacco [21]. In Tartary buckwheat, the expression of *FtMYB3* was induced by exogenous MeJA treatment and responded to cold stress by altering anthocyanin biosynthesis [22]. In rice, *OsMYB2*, *OsMYB3R-2*, *OsMYB4*, and *OsMYBS3* are also involved in modulating the cold-stress response [23,24,25]. 

MYB TFs can also participate in JA reaction processes, mainly by promoting the synthesis of anthocyanins and other substances to improve plant cold resistance. In JA-triggered signal transduction, the inhibitor JAZ in the SCFCOI1-JAZ ubiquitin proteasome degradation pathway plays a core role [26], and the MYB TFs interact with JAZ to mediate it [27,28]. The JAZ signaling pathway leads to the release of bHLH and MYB TFs, which promote anthocyanin accumulation in *Arabidopsis thaliana* [29]. In *Arabidopsis*, yeast two-hybrid assays indicated that MYB21 and MYB24 interact with JAZ 1/8/11 to mediate various jasmonate-regulated processes, including fertility, root growth, anthocyanin accumulation, senescence, and defense [30]. Wang et al. showed that MeJA can upregulate the expression of *MdMYB24*, which is involved in the regulation of JA-induced anthocyanin biosynthesis in apple [31]. *OsMYB30* negatively regulated *BMY* (beta-amylase) genes by interacting with *OsJAZ9* to fine-tune starch breakdown and maltose content, thereby improving the cold tolerance in rice [28]. In Tartary buckwheat, a yeast two-hybrid assay showed that *FtMYB3* interacted with *FtJAZ2*, playing a role in anthocyanin biosynthesis under cold stress [32]. An et al. showed that MdMYB9 interacted with MdbHLH3 and, additionally, that MdJAZ interacted with MdbHLH3 to promote anthocyanin biosynthesis [33]. MYB proteins form a JAZ-DELLA-MYBL2 complex that promotes the release of MYB/bHLH/WD40 and the formation of MBW complexes, thereby enabling the rapid accumulation of anthocyanins and improving plant cold tolerance [34]. Similarly, MdJAZ interacted with MdTRB1 (an apple-telomere-binding protein) and interfered with the interaction between MdTRB1 and MdMYB9, forming a JAZ1-TRB1-MYB9 module that dynamically modulated the JA-mediated accumulation of anthocyanin and proanthocyanidins (PAs) in apple [33]. *FtMYB18* mediated the inhibition of anthocyanin and PA synthesis by forming an MBW transcriptional complex with *FtTT8* and *FtTTG1* and an MYB-JAZ complex with FtJAZ1/-3/-4/-7 in Tartary buckwheat [35]. Taken together, these findings indicate that *JAZ* and *MYB* genes can directly or indirectly form a complex to respond to cold stress in plants.

MeJA is an efficient elicitor that can trigger the biosynthesis of volatile and nonvolatile secondary metabolites. Exogenous MeJA application has been widely used to improve the quality of horticultural crops. Examples of fruits with a flavor that has been enhanced after exogenous MeJA treatment include peach [36], strawberry [37], and loquat [38], and it has also effectively improved the aroma of tea [39,40]. Several previous studies have demonstrated that MeJA plays a crucial role in mediating plant resistance to pathogens [41,42,43]. The small-molecule isoquinoline compound ZINC 71820901 (lyn3) is an inhibitor of the JA signaling pathway, and it repressed tea plant resistance to *Ectropis grisescens*, mainly by reducing the accumulation of (-)epicatechin and (-)-epigallocatechin through repression of the JA signaling pathway [44]. Exogenous MeJA treatment increased PPO (Polyphenol Oxidase) activity by activating the transcription of CsPPO2 and CsPPO4, which mediate the JA signaling pathway and enhance tea resistance to the tea geometrid larvae (*Ectropis grisescens*) [45]. However, previous research has mainly focused on the effects of MeJA manipulation on tea aroma and insect resistance. Little is known about the molecular mechanisms by which MeJA treatment affects the response of tea to cold stress. This study firstly explores the mechanisms of JA signal transduction in response to cold stress in the tea plant and lays a theoretical foundation for further cultivation of cold-resistant tea-plant varieties.

## 2. Results

### 2.1. Content of Osmotic Adjustment Substances

**Figure 1:**
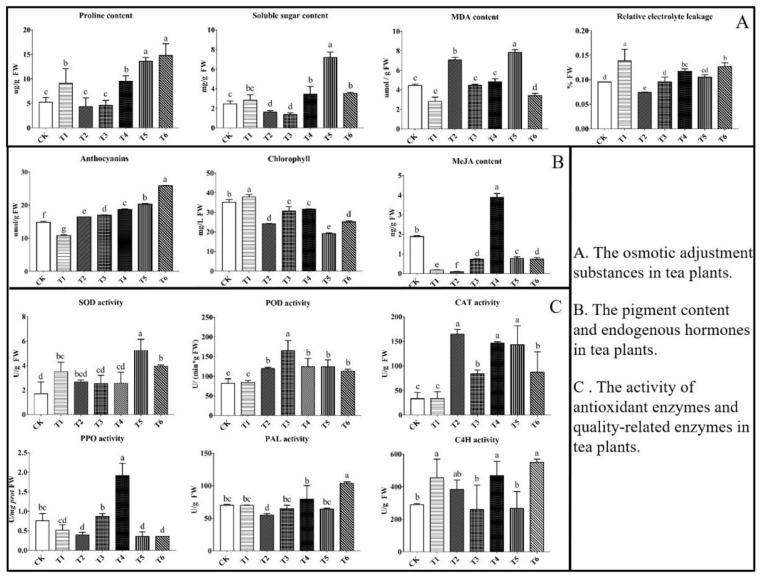
The effects of different treatments on resistance- and quality-related physiological indicators of tea plants. The error bars represent the standard error of the mean ± SD (*n* = 3). Different lowercase letters indicate significant differences at *p* < 0.05. Noted: MDA: malondialdehyde; MeJA: methyl jasmonate; SOD: Superoxide Dismutase; POD: Peroxidase; CAT: Catalase; PPO: Polyphenol Oxidase; PAL: Phenylalanineammonialyase; C4H: Cinnamate 4- Hydroxylase.

Four indicators, including the contents of proline, soluble sugar, malondialdehyde (MDA), and relative electrolyte leakage (REL), reflect the degree of membrane damage caused by cold stress (Figure 1A). Proline levels were significantly higher in T1, T4, T5, and T6 than in CK. The highest proline content was observed in T6 and increased as much as 2.83 times as that in CK. There were no significant differences in proline level between T2, T3, and CK. The soluble sugar content was significantly higher in T4, T5, and T6 than in CK. This showed the highest content in T5, which was about three times that of CK. MDA content was significantly increased in T2 but was decreased in T6 compared with CK. REL was significant higher in T1 and T6 than in CK, and, in addition, it was significantly higher in T5 and T6 than in T2. There were no differences between CK and the other treatments. 

### 2.2. Pigment Content and Endogenous MeJA Contents

In this study, we found that the anthocyanin content was significantly higher in T2, T3, T4, T5, and T6 than in CK. The anthocyanin content of T6 was 1.75 times higher than that of CK. However, the anthocyanin content of T1 was significantly lower than that of CK. Similarly, chlorophyll content was clearly higher in T1 than in CK but lower in the other five treatments. The endogenous MeJA content in T4 was 3.89 ng/g, significantly higher than that of CK (1.89 ng/g). In the other treatments, the endogenous MeJA content was clearly lower than that in CK. However, the endogenous MeJA contents in the T5 and T6 treatments were significantly higher than those in the T1 and T2 treatments, respectively (Figure 1B).

### 2.3. Activities of Antioxidant and Quality-Related Enzymes

The SOD activity of T5 was significantly higher than that of CK. The POD activity was significantly higher in T2, T3, T4, T5, and T6 than in CK and T1. Among of them, the POD activities under T3 exhibited a two-fold increase. CAT activity was significantly higher in T2, T3, T4, T5, and T6 than in CK and showed the highest value in T2. Polyphenol Oxidase (PPO) activity was significantly increased only in T4, in which it was 1.26 times higher than in CK. Our results indicated that PPO activity was significantly lower in T2, T5, and T6 than in CK. Phenylalanine ammonia lyase (PAL) activity was significantly higher in T6 and lower in T2 than in CK. Compared with CK, cinnamate 4-hydroxylase (C4H) activity was significantly higher in T1, T4, and T6 but showed no significant differences in T2, T3, and T5 (Figure 1C).

### 2.4. Expression Patterns of Sixteen MYB Genes

**Figure 2:**
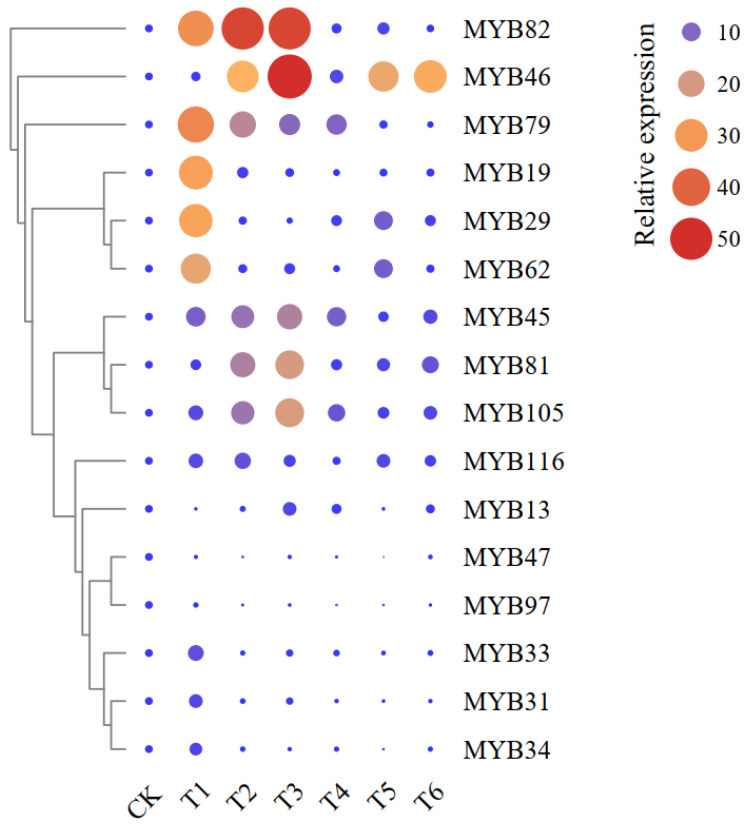
Relative expression levels of 16 *CsMYB* genes under different treatments. The circle sizes represent relative expression values of the *CsMYB* genes.

Transcriptome data derived from the Tea Plant Information Archive, for 127 R2R3-*MYB* genes in apical bud and young leaf tissues of the tea variety ‘Shuchazao’ in response to MeJA treatment and low temperature, were analyzed (Figure S1). Among the 127 *CsMYBs*, 16 were highly upregulated under cold-stress and MeJA treatment. Three key genes significantly differentially expressed under cold and MeJA treatments, *CsMYB45* was significantly expressed under cold stress alone (T1 and T2) and exogenous MeJA alone (T3 and T4) (*p* < 0.05). *CsMYB46* expression was significantly higher in T2, T3, T5, and T6 (combined cold-stress and exogenous MeJA treatments) than in CK (*p* < 0.05). Similarly, *CsMYB105* expression was significantly higher in T1, T2, T3, T4, and T6 than in CK (*p* < 0.05) (Figure 2). Compared to the other 13 CsMYB transcription factor genes, *CsMYB45*, *CsMYB46*, and *CsMYB105* showed significant upregulation in response to cold stress alone, exogenous MeJA alone, and the combined treatments. Their expression levels were highest in T3, suggesting 15, 54, and 21 times higher than those of CK, respectively. *CsMYB45*, *CsMYB46*, and *CsMYB105*, therefore, may have regulatory roles in the response to cold and exogenous MeJA treatments.

### 2.5. Cloning of CsMYB45, -46, and -105 and Subcellular Localization Assays

**Figure 3:**
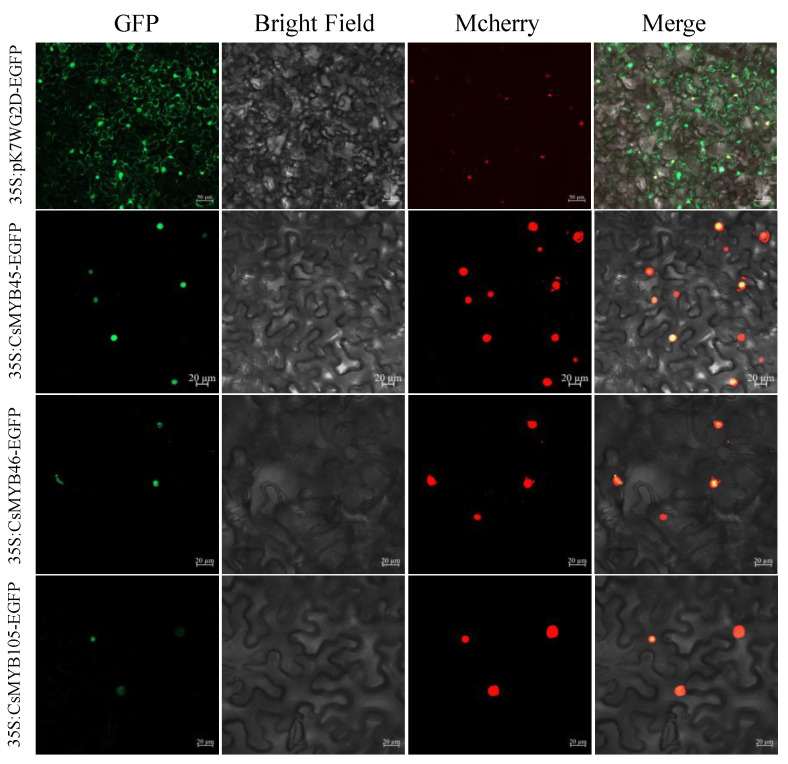
Subcellular localization of the fusion proteins 35S::*CsMYB45*::EGFP, 35S::*CsMYB46*::EGFP, and 35S::*CsMYB105*::EGFP expressed in tobacco epidermal cells by transient infiltration. The 35S::EGFP vector was used as the control, and P19-mCherry was used as a nuclear marker. Images are representative of at least three replicate experiments. Bar = 20 µm.

Subcellular localization of 35S::*CsMYB45*-EGFP, 35S::*CsMYB46*-EGFP, and 35S::*CsMYB105*-EGFP in tobacco epidermal cells showed that the three target genes were all located in the nucleus (Figure 3). This result was consistent with predictions of CsMYB45, CsMYB46, and CsMYB105 subcellular localization using the Softberry online tool.

### 2.6. Overexpression of CsMYB45, -46, and -105 in Escherichia coli

**Figure 4:**
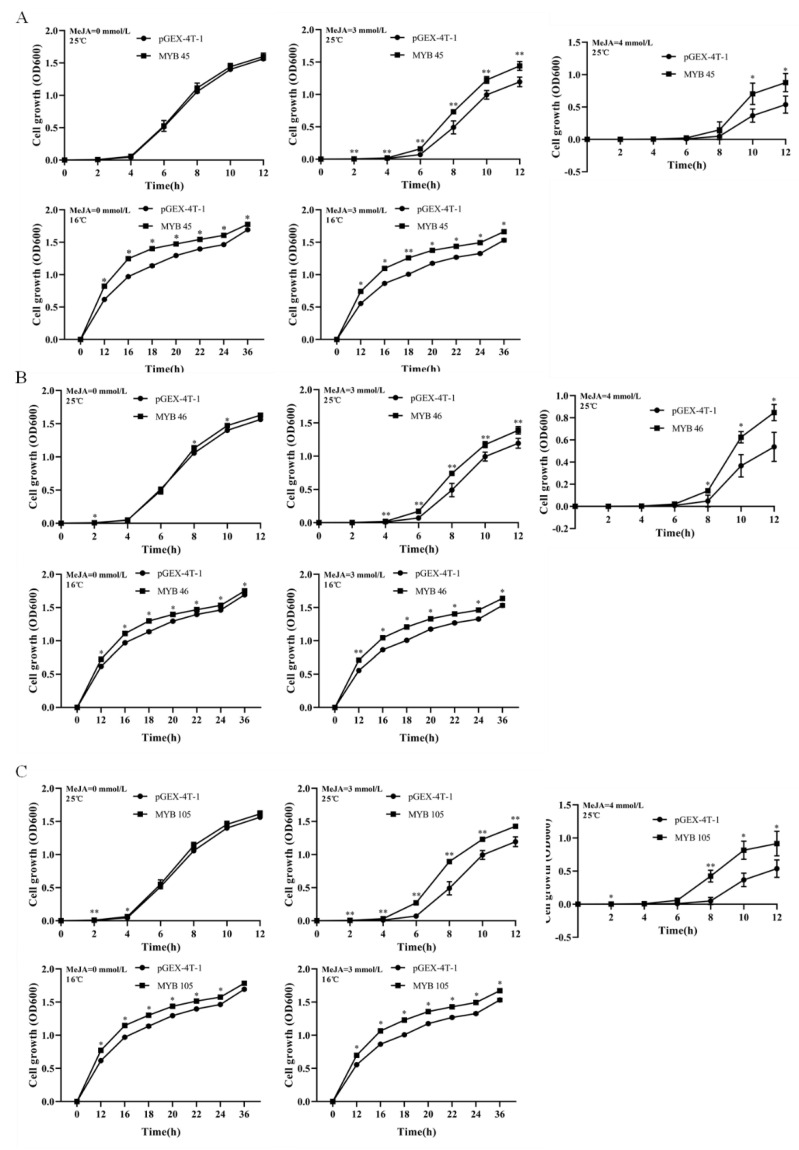
The growth rates of *E. coli* cells containing the overexpression vectors pGEX-4T-1-*CsMYB45* (**A**), pGEX-4T-1-*CsMYB46* (**B**), and pGEX-4T-1-*CsMYB105* (**C**) under cold stress alone (16 °C), MeJA treatment alone (3 or 4 mM), and the combined treatment. Data are shown as mean ± SD (*n* = 3). *, *p* < 0.05; **, *p* < 0.01.

The growth rates of *E. coli* cells that contained the overexpression vectors pGEX-4T-1-*CsMYB45*, pGEX-4T-1-*CsMYB46*, and pGEX-4T-1-*CsMYB105* did not differ from those of the empty vector control under normal conditions. However, under cold stress alone (16 °C), MeJA treatment alone (3 or 4 mM), and the combined treatment (16 °C cold stress and 3 mM MeJA), strains overexpressing *CsMYB45*, *CsMYB46*, and *CsMYB105* showed higher growth rates compared with the empty vector control (Figure 4). 

**Figure 5:**
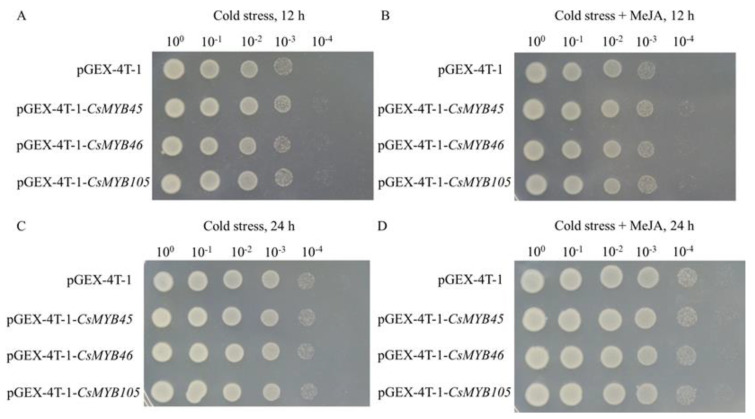
Spot assay of transgenic *E. coli* expressing pGEX-4T-1-*CsMYB45*, pGEX-4T-1-*CsMYB46*, pGEX-4T-1-*CsMYB105*, and pGEX-4T-1 (EV) on LB plates under cold stress at 16 °C for 12 h (**A**) and 24 h (**C**) and under the combined treatment with cold stress and exogenous MeJA for 12 h (**B**) and 24 h (**D**).

After 12 h, the pGEXT-4T-1 *E. coli* survival decreased rapidly, but the *CsMYB45*-, *CsMYB46*-, and *CsMYB105*-overexpressing cells were more viable than the control cell under cold stress (Figure 5A) and in the combined treatment (Figure 5B). After 24 h, the survival of the pGEXT-4T-1-*CsMYB* strains and the pGEXT-4T-1 cells did not differ significantly under cold stress alone (Figure 5C), but the pGEXT-4T-1-*CsMYB* strains cells showed better growth in the combined treatment (Figure 5D). These results indicate that overexpression of *CsMYB45*, *CsMYB46*, and *CsMYB105* can enhance the tolerance of *E. coli* strains to short-term cold stress. However, when the cold stress lasted for a longer time, the transgenic *E. coli* strains did not show any obvious resistance to cold stress alone. Therefore, we inferred that MeJA could improve the cold tolerance of strains overexpressing *CsMYB45*, *CsMYB46*, and *CsMYB105* in a short-term manner.

### 2.7. Transcriptional Activation and Yeast Two-Hybrid (Y2H) Assays of CsMYB45, -46, and -105

**Figure 6:**
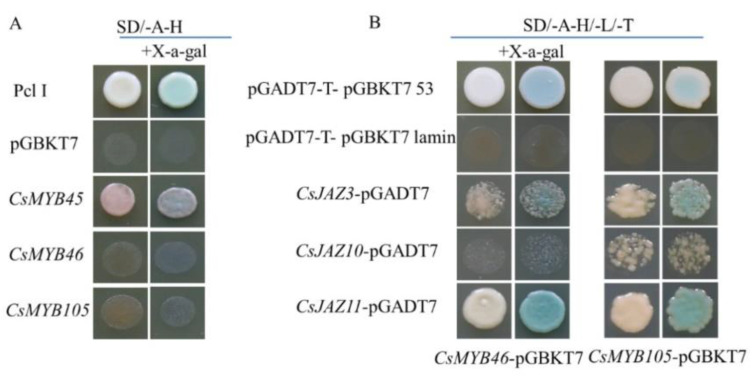

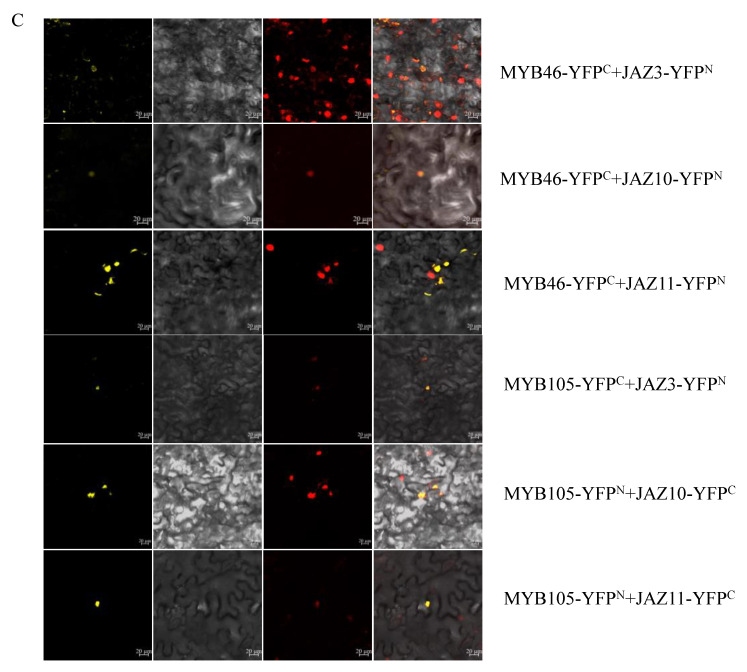
(**A**) Yeast self-activation activity assay of CsMYB45, CsMYB46, and CsMYB105. (**B**,**C**) Yeast two-hybrid assay (**B**) and BiFC assays (**C**) to screen for protein complex formation between the candidate CsMYBs and CsJAZ proteins. The interaction was verified by growth in SD/-Trp-Leu-His medium. The BiFC assay was performed in tobacco leaves. The MYB proteins were fused with the N-terminal part of YFP, and the CsJAZ proteins were fused with the C-terminal part of YFP. Images are representative of at least three replicate experiments. Bar = 50 µm.

Only CsMYB45 yeast cells exhibited β-galactosidase activity compared with the positive control pCL1 and the negative control pGBKT7. By contrast, yeast cells transformed with CsMYB46 or CsMYB105 did not show β-galactosidase activity, indicating that only CsMYB45 has self-activation activity (Figure 6A).

Yeast two hybrid assays indicated that CsMYB46 and CsMYB105 interacted with the inhibitor JAZ proteins CsJAZ3, CsJAZ10, and CsJAZ11 in the jasmonic acid signaling pathway (Figure 6B). The interactions among these TFs were further verified in tobacco (*N. benthamiana*) leaf cells by bimolecular fluorescence complementation (BiFC) assays, indicating that CsMYB-CsJAZ complexes are conserved in tea plants (Figure 6C).

## 3. Discussion

Tea plants are constantly subjected to various stresses, which will affect their production and final tea quality. Stresses can be alleviated by MeJA application, which is a plant-specific signaling molecule regulating a broad set of physiological and defense processes. MeJA can respond to cold stress through the regulatory networks of *JAZ* and *MYB* genes, which act as JA signaling pathway repressors. MYBs are essential for the control of plant development and defense responses toward biotic and abiotic stress. In this study, we found that spraying exogenous MeJA could alleviate cold stress damage with various degrees at the physiological, biochemical, and molecular levels and, therefore, improve the cold resistance of tea plants. 

### 3.1. Effect of MeJA on Osmotic Adjustment Substances in Tea Plants under Cold Stress

Previous study has now shown that the cold tolerance of tea plants is closely related to the contents of osmotic adjustment substances such as proline and soluble sugars [46]. Additionally, the cell membrane is the first barrier in plant cell defense against various environmental stresses. An increase in proline content helps plants maintain cell osmotic pressure, protecting the cell membrane system and alleviating the damage caused by cold stress. The proline content of tea plants decreased slightly after a long time of cold stress (T2) but increased in the combined treatments of cold stress and exogenous MeJA. Under MeJA treatment alone, proline content did not change significantly in T3 but was slightly higher in T4 compared with CK and T2. These results indicated that spraying exogenous MeJA can significantly increase proline content, tea plants show normal growth after cold stress, and a long time of spraying exogenous MeJA alone could also increase the proline content of tea plants. 

Soluble sugars are critical protective substances in plant cells. The accumulation of soluble sugars helps to regulate cellular osmotic pressure, decreasing the osmotic potential and the freezing point of water. We observed that the application of exogenous MeJA significantly increased soluble sugar content after tea plants were subjected to cold stress, which probably increased their self-repair ability and the formation of flavor substances under cold stress.

MDA and REL are two common physiological indicators that reflect the integrity of the cell membrane. MDA is the main metabolite of lipid peroxidation in the plant cell membrane, and REL reflects the permeability of the plant cell membrane to ions’ transportation, which will increase when membranes are damaged by stress. The values of these two indicators can reflect the degree of plant damage and provide an assessment of plant cold resistance. MDA accumulation increased significantly under cold stress in T2 but decreased significantly in T6, demonstrating that cold stress could cause membrane lipid peroxidation in the plant cell, and exogenous MeJA application could alleviate this damage. Based on the data from T5, spraying exogenous MeJA immediately after cold stress had no effect on improving the tolerance of tea plants. In addition, MDA content did not change significantly under MeJA treatment alone (T3 and T4). These results suggest that exogenous MeJA application can effectively enhance tea plant tolerance to cold stress. Under cold stress alone, REL increased remarkedly (T1), but there was no difference between CK and the T5 treatment when MeJA was applied together with cold stress. These results suggest that when tea plants were subjected to cold stress, the cell membrane structure was immediately damaged, resulting in increased permeability. However, exogenous MeJA application could effectively reduce REL under cold stress, assisting the tea plants’ return to their normal state. 

### 3.2. Effect of MeJA on Pigment Contents and Endogenous MeJA in Tea Plants under Cold Stress

Anthocyanins are the most oxidized flavonoids and have the ability to neutralize or scavenge reactive oxygen species (ROS) production under stress, thereby increasing the antioxidant capacity of plants and serving as a protective mechanism under low temperatures [47]. Here, we found that under cold stress alone, anthocyanin content was lowest in T1 but significantly higher than that of CK in T2. This result indicated that anthocyanin content decreased immediately after exposure to cold stress but increased again after the return of room temperature (25 °C) for a long time. This suggests that the tea plants can enhance their own defense capability by regulating their metabolism to increase the anthocyanin content. The anthocyanin content was significantly higher under MeJA treatment alone (T3 and T4) than under cold stress alone or CK conditions. These results indicated that the application of exogenous MeJA increased the accumulation of anthocyanins in tea plants, which were consistent with results from studies in strawberry and arabidopsis [9,48]. Furthermore, the anthocyanin content was significantly higher in the combined treatments than under cold stress or MeJA treatment alone, indicating that exogenous MeJA application can effectively enhance the cold resistance of the tea plant. 

Chlorophyll content increased immediately after cold stress alone (T1) but decreased after a long time of recovery from cold stress (T2). On the one hand, cold stress impairs the balance between chlorophyll synthesis and degradation, for example, by accelerating chlorophyll degradation and damaging the morphological structure of the chloroplast, thereby reducing chlorophyll content [49,50]. On the other hand, we speculate that anthocyanin content and chlorophyll content may be inversely correlated. These findings were similar to the physiological studies of cold tolerance in poplar and tobacco [51]. Here, we found that chlorophyll content was significantly lower in treatments with MeJA (T3, T4, T5, and T6) than in CK. Certain concentrations of MeJA can promote chlorophyll synthesis and increase the photosynthetic rate to facilitate the synthesis of photosynthetic products [52]. However, in this study, a relatively high concentration of exogenous MeJA (100 μmol/L) may increase the chlorophyll degradation and inhibit the synthesis of photosynthetic products, which is consistent with the results of Fan et al. [53]. In addition, Shahzad et al. (2015) showed that higher concentrations of exogenous MeJA strengthened plant defense, by limiting plant growth under stress conditions [54]. Plant hormones interact in biological signaling processes, including biosynthesis, metabolism, metabolite transportation, and the signal transduction pathways [55]. Under low-temperature stress, endogenous JA content increases rapidly and induces the synthesis of proteins or secondary metabolites related to stress defense, such as protease inhibitors, thereby establishing a plant defense system that inhibits reactive oxygen species and improving cold tolerance [56,57]. We found that the content of MeJA in T4 was doubled, indicating that the content of endogenous jasmonic acid did not increase immediately after spraying exogenous MeJA, though it increased sharply later. The content of endogenous MeJA was significantly higher in T5 and T6 (two combined treatments) than in T1 and T2 (cold stress treatment), indicating that the application of exogenous MeJA on tea plants under cold stress can increase the endogenous MeJA content and improve their cold tolerance. 

### 3.3. Effect of MeJA on the Activity of Antioxidant and Quality-Related Enzymes in Tea Plants under Cold Stress

Plants have evolved with complex mechanisms to combat the oxidative damage induced by ROS, including the activity increase in antioxidant enzymes such as SOD, POD, and CAT, which are positively correlated with plant cold resistance [58]. Here, we found that SOD activity was significantly higher in the combined treatments (T5), and there were no significant differences between the other treatments and CK. These results showed that cold stress or MeJA alone had no significant impact on SOD activity. However, exogenous MeJA application under cold stress could immediately increase SOD activity, thereby, presumably, improving cold resistance. POD and CAT activities were higher under exogenous MeJA treatment alone (T3 and T4) than under CK and T1, indicating that exogenous MeJA application could immediately increase POD and CAT activities. In addition, POD and CAT activities were significantly higher in T5 than in T1, suggesting that exogenous MeJA could effectively alleviate cold stress damage. In short, we found that exogenous MeJA promoted the activities of antioxidant enzymes under cold stress, and effectively mitigated the damage caused by cold stress to tea plants. Bagheri et al. suggested that the MeJA application reduces the MDA content and REL level and improves the antioxidant enzymes’ activities, leading to significant decreases in the chilling injuries of persimmon [59]. 

PPO plays a vital role in the development of tea buds, phenolic compounds synthesis, and the resistance of tea plants. It is particularly important for catalyzing the oxidation of polyphenols to quinones during tea processing [60]. In this study, PPO activity was significantly higher in T4 (MeJA treatment alone) than in CK, indicating that exogenous MeJA could markedly increase the PPO activity, which is consistent with the study of Zhou et al. [61]. According to Wang Li et al., the potato *PPO* gene family members *StuPPO1*, *StuPPO2*, *StuPPO3*, *StuPPO6*, *StuPPO8*, and *StuPPO9* contain MeJA-responsive elements [62]. Therefore, it is speculated that *PPO* may be involved in the regulation of phenolic synthesis in response to MeJA, and low-temperature stress could inhibit the PPO activity.

PAL and C4H are essential enzymes in the phenylpropanoid biosynthetic pathway. Trans-coumaroyl-CoA, a product of this pathway, was involved to generate anthocyanins and anthocyanidins through the flavonoid pathway, thereby participating in plant responses to abiotic stresses. PAL is considered to be the gateway for the phenylpropanoid pathway [63]. Here, PAL content was higher in T4 and T6 than in CK and T2, indicating that exogenous MeJA application increased PAL activity, which may prevent cold damage to tea plants. Similarly, C4H activity was significantly higher in T4 and T6 than in CK, showing that exogenous MeJA also markedly increased the C4H activity. We, therefore, found that exogenous MeJA application induced the activities of PPO, PAL, and C4H in tea plants under normal conditions and cold stress, which would presumably promote the synthesis and accumulation of essential secondary phenylpropanoid metabolites such as lignin, flavonoids and anthocyanins and, therefore, enhance the stress resistance of tea plants.

### 3.4. Identification and Functional Verification of Key CsMYB Genes

MYB proteins, particularly R2R3-MYB TFs, have been found to regulate the networks that control diverse plant processes, including responses to biotic and abiotic stresses, development, and primary and secondary metabolism [64]. Although, a previous study showed that 122 R2R3-MYB TFs have been identified in tea plants [19]. However, in this study, gene expression for MYB TFs under MeJA and cold stress treatments were obtained from the genome database of the tea plant variety ‘Shuchazao’, and a total of 127 MYB TFs were identified. Sixteen showed high expression under cold stress and MeJA treatment, and their expressions were verified by qRT-PCR under individual and combined cold stress and MeJA treatments. Three *CsMYB* genes showed strong upregulation in response to these treatments. *CsMYB45* expression increased significantly in response to exogenous MeJA alone and low-temperature stress alone. *CsMYB46* and *CsMYB105* expression levels were markedly higher under the individual and combined treatments than under control conditions. We, therefore, speculated that these three CsMYB TFs may play an essential role in the response to low-temperature stress via the JA signaling pathway. 

CsMYB45, CsMYB46, and CsMYB105 were localized in the nucleus of tobacco epidermal cells, which was consistent with previous findings [65,66]. Relevant studies have shown that the growth of *E. coli* cells’ overexpressing genes with interest is one of the methods to validate gene function [67,68]. Here, we found that overexpression of *CsMYB45*, *CsMYB46*, and *CsMYB105* enhanced the tolerance of *E. coli* to low-temperature stress. Strains overexpressing *CsMYBs* under cold stress alone (16 °C), MeJA treatment alone, or the combined treatment showed better growth rates compared with the empty vector and normal conditions (25 °C). We also observed that strains overexpressing *CsMYB45*, *CsMYB46*, and *CsMYB105* showed enhanced cold tolerance when exogenous MeJA was added. These results prove that *CsMYB45*, *CsMYB46*, and *CsMYB105* play essential roles in the cold stress response of *E. coli* via the jasmonic acid signal transduction pathway.

MYB family genes have been shown to regulate the jasmonic acid signal transduction pathway by interacting with the inhibitor JAZ in the SCFCOI1-JAZ ubiquitin proteasome degradation pathway. They participate in a various of jasmonic acid related processes, including JA-induced anthocyanin synthesis and the regulation of plant cold resistance. A previous study revealed that *Arabidopsis* MYB proteins interacted with several JAZ proteins and formed dimers [27]. For example, Arabidopsis AtMYB21 and AtMYB24 interact with JAZ1, JAZ8, and JAZ11 in yeast two-hybrid assays [30]. A report by Luo et al. suggests that FtJAZ2 interacts with FtMYB3 under cold stress and participates in the biosynthesis of anthocyanins in Tartary buckwheat [32]. Furthermore, the interaction between JAZ1 and WD-Repeat/bHLH/MYB complexes regulates jasmonate-mediated anthocyanin accumulation and trichome initiation in *Arabidopsis* [34]. According to previous report, sequence alignment results showed that CsJAZ3, CsJAZ10, and CsJAZ11 were AtJAZ1-like proteins, all of which are responsive to exogenous MeJA treatment [67]. Therefore, we used yeast two-hybrid and bimolecular fluorescence complementation (BiFC) assays to provide preliminary evidences that CsMYB46 and CsMYB105 interact with the inhibitor JAZ proteins CsJAZ3, CsJAZ10, and CsJAZ11 in the jasmonic acid signaling pathway.

## 4. Material and Methods

### 4.1. Plant Material and Treatments

One-year-old cuttings of the tea plant cultivar ‘Longjing43’ were preincubated in an artificial climate chamber at the Tea Science Research Institute of Nanjing Agricultural University (Nanjing, China), where the photoperiod was 8 h light (25 °C)/16 h dark (20 °C) with a light intensity of 200 μmol m^−2^ s^−2^ and 75% relative humidity for one week. For the cold stress treatments, tea plants were exposed to 4 °C for 4 days. For exogenous MeJA treatments, tea plants were sprayed with 100 μM MeJA on 4 consecutive days. These treatments were combined to produce six experimental treatment regimens (Table 1). 

Three biological replicates, consisting of one bud and two leaves in each, were collected from the six treatments and the control. All samples were immediately frozen in liquid nitrogen and stored at −80 °C for subsequent experiments. 

### 4.2. Physiological and Biochemical Analysis of Tea Plants

#### 4.2.1. Pigment Contents

Total anthocyanin content was measured according to the Proctor [69] method. Each freeze-dried and crushed sample (0.1 g) was extracted by 10 mL of 0.1 M HCl in ethanol (4 mL HCl and 476 mL ethanol) and incubated in 60 °C water bath for 30 min with intermittent shaking (10 s on vortex mixer). The extract solution was filtered, and its absorbance was read at 530, 620, and 650 nm using an UV–Vis spectrophotometer (P4, MAPADA, Shanghai, China). Anthocyanin content was calculated based on the formula: △A = (A530 − A620) − 0.1 (A650 − A620) [69]. Total anthocyanin contents were calculated according to the following formula: total anthocyanin (μmol/g) = (△A × 100)/(4.62 × sample weight) (A represent absorbance). 

Chlorophyll contents were determined using an ultraviolet spectrophotometer, following the method described by Lichtenthaler [70]. Each tea plant sample (0.2 g) was ground into homogenate with 80% acetone on ice. The mixture was extracted overnight at room temperature in the dark until the plant material changed to colorless. Plain 80% acetone was used as the control solution, and absorbance was read at 663 nm, 652 nm, and 645 nm. Chlorophyll contents were calculated using the following formulas:

Chlorophyll a (mg/g) = (12.72A663 − 2.59A645) × Vs/1000 w

Chlorophyll b (mg/g) = (22.88A645 − 4.67A663) × Vs/1000 w

where Vs is the total volume of the extract solution, W is the sample fresh weight.

All analyses were repeated three times.

#### 4.2.2. Relative Ion Leakage Rate

The relative ion leakage rate was measured by referring to Cai et al. [71]. Leaf disks were punched from randomly selected samples of fresh, healthy, and intact tea leaves, avoiding the major leaf vein areas. Ten randomly selected disks were placed in a 10 mL tube with 10 mL of deionized water; five replicate tubes were used for each treatment. After filtering under a vacuum pump for 30 min, we measured the initial conductivity of the solution (C1). The tubes were then boiled in deionized water for 10 min, and the conductivities of the resulting solutions (C2) were measured after the tubes cooled to 25 °C. The relative ion leakage rate was calculated as (C1/C2) × 100%.

#### 4.2.3. Other Physiological and Biochemical Measurements

Proline, MDA, and soluble sugar contents were measured as described by Cai et al. [71]. POD (Peroxidase), CAT (Catalase), SOD (Superoxide Dismutase), PPO (Polyphenol Oxidase), PAL (Phenylalanineammonialyase), and C4H (Cinnamate 4- Hydroxylase) activities were determined using enzyme activity kits (Jiancheng, Nanjing, China), in accordance with the instructions of the manufacturer. Endogenous JA content was determined by Nanjing Jisihuiyuan Biotechnology Co., Ltd. (Nanjing, China).

### 4.3. Identification of MYB Genes in Camellia sinensis

Based on the Tea Plant Information Archive database (TPIA, http://tpia.teaplant.org/index.html (accessed on 10 May 2019)), 127 MYB TF family members related to MeJA and cold stress were identified in the tea plant cultivar ‘Shuchazao’. Through heat map analysis of the expression levels of these 127 *MYB* genes, in response to low temperature and MeJA, 16 *MYB* genes with high expression under low temperature and MeJA treatment were identified, and their gene sequences and the expression data were obtained from the Tea Plant Genome Database (http://tpia.teaplant.org/download.html (accessed on 10 May 2019)). 

### 4.4. Total RNA Extraction and Quantitative Real-Time PCR

Total RNA was isolated from the samples using the RNA Quick Isolation kit (Aidlab, Beijing, China), and reverse transcription was performed with One-Step gDNA Removal and cDNA Synthesis SuperMix (TransGen, Beijing, China). Expression analysis of the sixteen *CsMYB* genes was performed by quantitative real-time PCR using the SYBR Premix Ex Taq II kit (Takara, Kusatsu, Japan). Csβ-actin gene was used as an internal control. The primer pairs used for qRT-PCR were listed in Table S1. The qRT-PCR program was as follows: 95 °C for 30 s, followed by 40 cycles of 95 °C for 5 s and 60 °C for 30 s. All reactions were performed with three biological and technical replicates. The relative gene expression levels of the *CsMYB* genes were calculated using the 2^−ΔΔCt^ method [72]. Based on this result, three key *CsMYB* genes were identified to show significantly different expression under the cold and MeJA treatments, which were used in the following experiments.

### 4.5. Subcellular Localization Assays for CsMYB45, -46, and -105

The *CsMYB45*, *CsMYB46*, and *CsMYB105* ORF sequence were amplified from the entry vector pDONR201 construct using the pDONR-attb-*CsMYBs* -F/R primer pair (Table S1) harboring via BP reaction (Invitrogen, Carlsbad, CA, USA). The vector pDONR201-*CsMYBs* was recombined with the expression vector pK7FWG2.0-D via LR reaction (Invitrogen, Carlsbad, CA, USA) to generate fusion vectors 35S::*CsMYB45*-EGFP, 35S::*CsMYB46*-EGFP, and 35S::*CsMYB105*-EGFP. The recombinant plasmid (35S::*CsMYB45*-EGFP, 35S::*CsMYB46*-EGFP, and 35S::*CsMYB105*-EGFP) and the empty vector 35S::EGFP were transformed into cells of *Agrobacterium tumefaciens* strain GV3101. Subsequently, we co-transformed *GV3101* containing the recombinant plasmids or empty vector with the nuclear marker P19-mCherry for transient transformation in tobacco leaves (*Nicotiana benthamiana*). Plants were incubated in the dark for 48 h after infection, and leaves’ GFP fluorescence signal was monitored with Zeiss LSM 800 confocal microscope (Carl Zeiss AG, Oberkochen, Germany). 

### 4.6. Prokaryotic Expression Assay of CsMYB45, -46, and -105 

The open reading frames (ORFs) of *CsMYB45*, *CsMYB46*, and *CsMYB105* were digested with *EcoRI*, and the resulting gene fragments were cloned into the expression vector pGEX-4T-1 (Amersham Biosciences) using Cloning Kit C112 (Vazyme Biotech Co., Ltd. Nanjing, China) (Table S1). The recombinant plasmid (pGEX-4T-1-*CsMYB45*, pGEX-4T-1-*CsMYB46*, and pGEX-4T-1-*CsMYB105*) and the empty vector pGEX-4T-1 were transformed into *Escherichia coli ROSETTA* cells and cultured in LB medium with 100 mg/L ampicillin at 37 °C until the optical density at 600 nm (OD600) reached 1.0. The transformants harboring and empty strain were added to LB medium (100 mg/L ampicillin), containing different concentrations of MeJA (0 mM, 3 mM, and 4 mM) at a ratio of 1:1000. Each mixture was then divided into two parts: one was placed in a constant 37 °C temperature incubator with shaking at 200 rpm, and the OD600 value was determined with an ultraviolet spectrophotometer after different incubation times (0 h, 2 h, 4 h, 6 h, 8 h, 10 h, and 12 h).

The other part was exposed to low temperature (16 °C) at an initial OD600 value for the four strains set to 0.2 with shaking at 200 rpm. Cold stress could inhibit the growth rate of *E. coli*, so the incubation times was prolonged. The OD600 value was measured with an ultraviolet spectrophotometer at different incubation times (0 h, 12 h, 16 h, 20 h, 24 h, 36 h, and 48 h). Strains exposed to different treatments with different culture durations were then collected and inoculated onto LB basal plates containing 100 g/L ampicillin. All plates were cultured at 37 °C overnight for 12 h, and the growth of *E. coli* was observed and photographed.

### 4.7. Transcriptional Activation Assay of CsMYB45, -46, and -105

The transcriptional activation of CsMYB45, CsMYB46, and CsMYB105 was determined in the yeast system. The ORFs of CsMYB45, CsMYB46, and CsMYB105 were constructed in the pGBKT7 vector using *BamHI*, resulting in the plasmids pGBKT7-*CsMYB45*, pGBKT7-*CsMYB46*, and pGBKT7-*CsMYB105*. pCL1 (positive control), and pGBKT7 (negative control) plasmids were introduced into *Saccharomyces cerevisiae strain* Y2H Gold (Clontech), in accordance with the protocol of the manufacturer (Table S1). The transformants harboring the pGBKT7-*CsMYB45*, pGBKT7-*CsMYB46*, and pGBKT7-*CsMYB105* or pGBKT7 plasmids were selected on SD/-Trp medium (SD, Synthetic Dropout), whereas those harboring pCL1 were selected on SD/-Leu medium. As a positive control, Y2H cells harboring pCL grew well on SD/-His-Ade medium, but the negative control Y2H cells harboring pGBKT7 did not grow on SD/-His-Ade medium. 

### 4.8. Yeast Two-Hybrid (Y2H) and BiFC Assay 

In this study, we identified the interaction relationship of 3 CsJAZ proteins obtained in previous studies with the pGBKT7-*CsMYB45*, pGBKT7-*CsMYB46*, and pGBKT7-*CsMYB105*, and a verification experiment was performed using Clontech system (Clontech, Mountain View, CA, USA). The positive control plasmids were pGADT7-T and pGBKT7-53, and the negative control plasmids were pGADT7-T and pGBKT7-lam. A construct of the ORF of *CsJAZ3*, *CsJAZ10*, and *CsJAZ11* in the pGADT7 vector was generated using *BamHI*, resulting in the plasmids pGADT7-JAZ3, pGADT7-JAZ10, and pGADT7-JAZ11, with the CsJAZ3, CsJAZ10 and CsJAZ11-AD-F/R primers (Table S1). All plasmids were transferred into the Y2H yeast strain (Clontech). The growth of transformed yeast cells was test on SD/-Trp-Leu medium, SD/-Trp-Leu-His-Ade medium, and SD/-Trp-Leu-His-Ade medium containing X-α-Gal. The transformed yeast cells were incubated at 30 °C for 3–5 days, and the monoclonal clones that turned blue were selected for PCR detection and photographed.

Bimolecular fluorescence complementation (BiFC) assays were performed in tobacco epidermis cells. The ORFs of *CsMYB45*, *CsMYB46* and *CsMYB105* was introduced into the pCAMBIA 1300-35S vector containing the C-terminal of YFP to generate pCAMBIA 1300-35S-C-YFPC-*CsMYBs* construct via *BamHI* restriction enzymes. The ORF of CsJAZ3, CsJAZ10, and CsJAZ11 were fused with the N-terminal fragment of the YFP protein in the pCAMBIA 1300-35S vector, after restriction digestion with *BamHI*, to produce the pCAMBIA 1300-35S-C-YFPN-CsJAZs construct. The constructs were separately transformed into *A.tumefaciens* strain GV3101 and then con-reansformed into the leaves of 4-week-old *Nicotiana benthamiana* plants. After culturing in the dark for 1 d and in light for 2 d, the tobacco epidermal cells were observed with a confocal laser scanning microscope (LSM 800, Carl Zeiss AG, Oberkochen, Germany). The primers used for vector construction are shown in Table S1.

### 4.9. Statistical Analyses

All data in this study were analyzed using Excel (version 2018) and IBM SPSS Statistics (version 26.0), and the results were presented as the mean ± standard deviation (SD). The data were analyzed in Excel, and the visualizations were analyzed with GraphPad Prism software (version 8.0). Significant differences between the experimental treatments were tested by SPSS (version 26.0) using one-way ANOVA, and Duncan’s multiple range test was used as indicated by different letters (*p* < 0.05).

## 5. Conclusions

In summary, physiological and biochemical assays showed that exogenous MeJA application could effectively promote ROS scavenging in the tea plant under cold stress, maintain the stability of the cell membrane, and further relieve the damage caused by cold stress, thereby enhancing the cold tolerance of tea plants. Sixteen of 127 *CsMYB* genes showed high relative expression levels in response to MeJA or cold-stress treatments. Three *CsMYB* genes were strongly induced under a combination of MeJA and cold-stress treatment. Subcellular localization assays demonstrated that CsMYB45, CsMYB46, and CsMYB105 fused proteins were localized in the cell nucleus. Exogenous MeJA treatment enhanced the overexpression of *CsMYB45*, *CsMYB46*, and *CsMYB105* in *E. coli* and improved the growth and survival rates of recombinant cells under cold stress. Yeast two-hybrid and bimolecular fluorescence complementation experiments confirmed that CsMYB46 and CsMYB105 interacted with CsJAZ3, CsJAZ10, and CsJAZ11 in the nucleus. These findings extend our understanding of how MYB TFs participate in the regulation of cold tolerance via the jasmonic acid pathway and provide a theoretical foundation for future functional studies.

## Figures and Tables

**Table 1:** Descriptions of control and six experimental treatments applied to tea plants.

Treatment	Days 1–4	Days 5–8	Day 9
CK	Room temperature and water spray	Room temperature and water spray	Samples collected
T1	Room temperature and water spray	4 °C cold stress	Samples collected
T2	4 °C cold stress	Room temperature and water spray	Samples collected
T3	Room temperature and water spray	Room temperature and 100 μM MeJA spray	Samples collected
T4	Room temperature and 100 μM MeJA spray	Room temperature and water spray	Samples collected
T5	Room temperature and water spray	4 °C cold stress and 100 μM MeJA spray	Samples collected
T6	4 °C cold stress and 100 μM MeJA spray	Room temperature and water spray	Samples collected

## Data Availability

Not applicable.

## Supplementary Materials

The following are available online at https://www.mdpi.com/article/10.3390/plants11212869/s1. Figure S1: Expression patterns of MYB genes in response to MeJA and cold treatments. Table S1: Primer information. Ref. [73] has been cited in supplementary materials.
