# Supplementary material for: CsMYB Transcription Factors Participate in Jasmonic Acid Signal Transduction in Response to Cold Stress in Tea Plant (Camellia sinensis)
Source: Plants (Basel). 2022 Oct 27;11(21):2869. doi: 10.3390/plants11212869 (PMC9654479; doi:10.3390/plants11212869)
Supplement: Supplementary file 1 [file plants-11-02869-s001.zip › plants-1984337-supplementary.pdf]

Table S1. Primer information.

Primer Name	Primer Sequence
**Primer for qRT-PCR**	
Csβ-actin-q-F	GCCATCTTTGATTGGAATGG
Csβ-actin-q-R	GGTGCCACAACCTTGATCTT
CsMYB13-q-F	AGGCTACCAGGGCGAACAG
CsMYB13-q-R	TTTGGCTCGGACCACATCA
CsMYB19-q-F	AACCTCAGCCAGAAATGC
CsMYB19-q-R	AACTCACAAGGCGTCCAG
CsMYB29-q-F	CCTTGAGTGAGTTGGGTGAT
CsMYB29-q-R	CCAGCATTCTTCGGCATA
CsMYB31-q-F	ACAGACAAACCCGGCAAAC
CsMYB31-q-R	TGGTGACCTGGAGGAAGC
CsMYB33-q-F	ACAAGGAAGGTGAGGGACG
CsMYB33-q-R	GTTGCCGAGAAGGCGATG
CsMYB34-q-F	CGCTGCGGAAAGAGTTGC
CsMYB34-q-F	TGGAGGCGGAGAATGAGG
CsMYB45-q-F	CTCCCTCACCCTATCCTTACCC
CsMYB45-q-R	CACCACTGCGTTCCTAATCG
CsMYB46-q-F	GGAACCGACCCGAATACC
CsMYB46-q-R	TTGAGGAAGAACCGCCAC
CsMYB47-q-F	GTCAGGGTTGCGTAGATG
CsMYB47-q-R	GGAGATTCGAGGACAGAAG
CsMYB62-q-F	GGACGAACCGACAACGAAA
CsMYB62-q-R	TGCTGGTGGTGGTGATGGA
CsMYB79-q-F	ATTATCCAGTCACGGCCCATCC
CsMYB79-q-R	CCGCCATTCCAAGCATTTAGT
CsMYB81-q-F	GGGAGATTGCCAGGACGAA
CsMYB81-q-R	CACGGTCTTTAGAAGCAGATGAG
CsMYB82-q-F	ATCTCCGTCCAGGTATCAA
CsMYB82-q-R	AACCCAAATCAGGAAATCG
CsMYB97-q-F	ATGATTGGTGGGATGAGC
CsMYB97-q-R	TAGGATGGACCAGCAAAG
CsMYB105-q-F	GGTGGTCCTTGATTGCTG
CsMYB105-q-R	GGCGTGCTTTCTTGTTCC
CsMYB116-q-F	CCCAAGTCCCTTCTTTATCTC
CsMYB116-q-R	TATTTCCATCCTAAGCCAACG
**Primer for gene cloning**	
CsMYB45-F	ATGGCTTCTTTGTCTAGGAAGGA
CsMYB45-R	CTCGATCTTGCTAATCCCAATTC
CsMYB46-F	ATGGGAAGGGCTCCATGTT
CsMYB46-R	TATCAGCAAAGATTCAGCAAAG
CsMYB105-F	ATGGCTCCGAAGAGCAGT
CsMYB105-R	CCATTTATCGGTAAGTGCC
**Primer for subcellular localization assays**	
CsMYB45-attb-F	GGGGACAAGTTTGTACAAAAAAGCAGGCTTCATGGCTTCTTTGTCTAGGA AGGA
CsMYB45-attb--R	GGGGACCACTTTGTACAAGAAAGCTGGGTCCTCGATCTTGCTAATCCCAA TTC
CsMYB46-attb--F	GGGGACAAGTTTGTACAAAAAAGCAGGCTTCATGGGAAGGGCTCCATGTT
CsMYB46-attb--R	GGGGACCACTTTGTACAAGAAAGCTGGGTCTATCAGCAAAGATTCAGCAA AG
CsMYB105-attb--F	GGGGACAAGTTTGTACAAAAAAGCAGGCTTCATGGCTCCGAAGAGCAGT GA
CsMYB105-attb--R	GGGGACCACTTTGTACAAGAAAGCTGGGTCCCATTTATCGGTAAGTGCCT GAT
**Primer for prokaryotic expression assay**	

pGEX-4T-1-*CsMYB45*-F	CGCGTGGATCCCCGGAATTCATGGCTTCTTTGTCTAGGAAGGA
pGEX-4T-1-*CsMYB45*-R	GCTCGAGTCGACCCGGGAATTCCTCGATCTTGCTAATCCCAATTC
pGEX-4T-1-*CsMYB46*-F	CGCGTGGATCCCCGGAATTCATGGGAAGGGCTCCATGTT
pGEX-4T-1-*CsMYB46*-R	GCTCGAGTCGACCCGGGAATTCTATCAGCAAAGATTCAGCAAAG
pGEX-4T-1-*CsMYB105*-F	CGCGTGGATCCCCGGAATTCATGGCTCCGAAGAGCAGTGA
pGEX-4T-1-*CsMYB105*-R	GCTCGAGTCGACCCGGGAATTCCCATTTATCGGTAAGTGCCTGAT
**Primer for yeast two-hybrid and BiFC assay**	
CsMYB45-BD-F	AGGCCGAATTCCCGGGGATCCATGGCTTCTTTGTCTAGGAAGGA
CsMYB45-BD-R	CCGCTGCAGGTCGACGGATCCCTCGATCTTGCTAATCCCAATTC
CsMYB46-BD-F	AGGCCGAATTCCCGGGGATCCATGGGAAGGGCTCCATGTT
CsMYB46-BD-R	CCGCTGCAGGTCGACGGATCCCCATTTATCGGTAAGTGCCTGAT
CsMYB105-BD-F	AGGCCGAATTCCCGGGGATCCATGGCTCCGAAGAGCAGTGA
CsMYB105-BD-R	CCGCTGCAGGTCGACGGATCCCCATTTATCGGTAAGTGCCTGAT
CsJAZ3-AD-F	GTGGGCATCGATACGGGATCCATGTCGAGTTCGTCTGGT
CsJAZ3-AD-R	CAGCTCGAGCTCGATGGATCC CAAATGGTGCTCAAACTGC
CsJAZ10-AD-F	GTGGGCATCGATACGGGATCCATGTCGAGTTCGTCTGGTTC
CsJAZ10-AD-R	CAGCTCGAGCTCGATGGATCCCCTATCTTTTCTCTTCTCCA
CsJAZ11-AD-F	GTGGGCATCGATACGGGATCCATGGCAAGTTCTCAAGTGTT
CsJAZ11-AD-R	CAGCTCGAGCTCGATGGATCCCAACTTGAGATCAAGCTGTT
pCAMBIA 1300-35S-C-YFPC-*CsMYB46*-F	GAGCTCGGTACCCGGGGATCCATGGGAAGGGCTCCATGTT
pCAMBIA 1300-35S-C-YFPC-*CsMYB46*-R	CATGTCGACTCTAGAGGATCCTATCAGCAAAGATTCAGCAAAG
pCAMBIA 1300-35S-C-YFPC-*CsMYB105*-F	GAGCTCGGTACCCGGGGATCCATGGCTCCGAAGAGCAGTGA
pCAMBIA 1300-35S-C-YFPC-*CsMYB105*-R	CATGTCGACTCTAGAGGATCCCCATTTATCGGTAAGTGCCTGAT
pCAMBIA 1300-35S-C-YFPN-*CsJAZ3*-F	GAGCTCGGTACCCGGGGATCCATGTCGAGTTCGTCTGGT
pCAMBIA 1300-35S-C-YFPN-*CsJAZ3*-R	CATGTCGACTCTAGAGGATCCCAAATGGTGCTCAAACTGC
pCAMBIA 1300-35S-C-YFPN-*CsJAZ10*-F	GAGCTCGGTACCCGGGGATCCATGTCGAGTTCGTCTGGTTC
pCAMBIA 1300-35S-C-YFPN-*CsJAZ10*-R	CATGTCGACTCTAGAGGATCCCCTATCTTTTCTCTTCTCCA
pCAMBIA 1300-35S-C-YFPN-*CsJAZ11*-F	GAGCTCGGTACCCGGGGATCCATGGCAAGTTCTCAAGTGTT
pCAMBIA 1300-35S-C-YFPN-*CsJAZ11*-R	CATGTCGACTCTAGAGGATCCCAACTTGAGATCAAGCTGTT

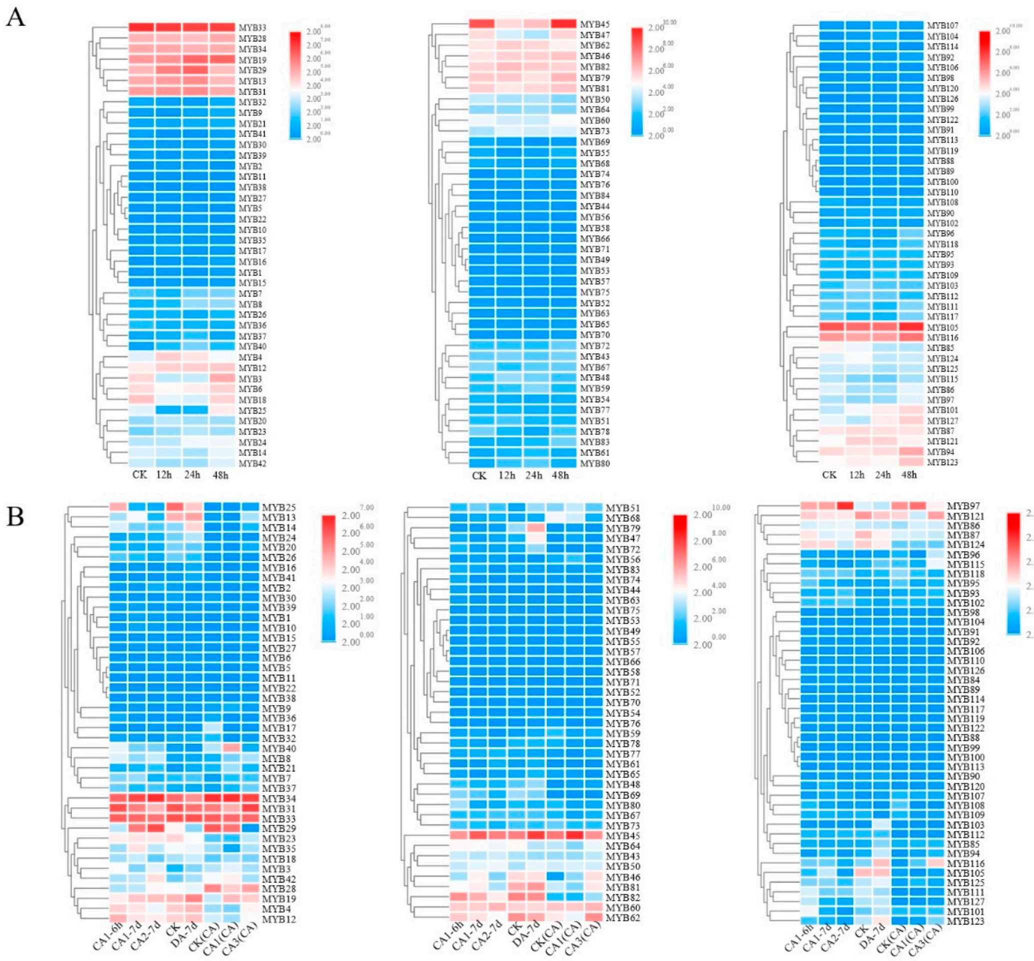

Figure S1. Expression patterns of *MYB* genes in response to MeJA and cold treatments. A. Gene expression at different time points after MeJA treatment. B. Gene expression at three time periods during the cold acclimation (CA) process, including different periods of CA: non-acclimated (25–20 °C, CK), fully acclimated at 10 °C for 6 h (CA1-6h) and at 10–4 °C for 7 days (CA1-7d), cold response at 4–0 °C for 7 days (CA2-7d), recovery at 25–20 °C for 7 days (DA-7d), and non-acclimated (CK), fully acclimated (CA1), and de-acclimated (CA3)[1].

Reference

1. Wang, X. C., Zhao, Q. Y., Ma, C. L., Zhang, Z. H., Cao, H. L., Kong, Y. M., Yue, C., Hao, X. Y., Chen, L., Ma, J. Q., Jin, J. Q., Li, X. & Yang, Y. J. Global transcriptome profiles of Camellia sinensis during cold acclimation. Bmc Genomics 14, doi:10.1186/1471-2164-14-415 (2013).
